# ﻿A striking color variation is detected in *Poneratestacea* Emery, 1895 (Hymenoptera, Formicidae) across its Western Palaearctic geographic range

**DOI:** 10.3897/zookeys.1084.79415

**Published:** 2022-02-01

**Authors:** Sándor Csősz, Kadri Kiran3, Celal Karaman3, Albena Lapeva-Gjonova

**Affiliations:** 1 Evolutionary Ecology Research Group, Institute of Ecology and Botany, Centre for Ecological Research, Alkotmány út 2-4, Vácrátót H-2163, Hungary Evolutionary Ecology Research Group, Institute of Ecology and Botany Budapest Hungary; 2 MTA-ELTE-MTM Ecology Research Group, Pázmány Péter sétány 1/C, Budapest H-1117, Hungary MTA-ELTE-MTM Ecology Research Group Budapest Hungary; 3 Trakya University, Faculty of Science, Department of Biology, Edirne, Turkey Trakya University Edirne Turkey; 4 Sofia University, Faculty of Biology, Department of Zoology and Anthropology, Bulgaria Sofia University Sofia Bulgaria

**Keywords:** Biogeography, exploratory analyses, gap statistic, morphometry, species delimitation

## Abstract

In this paper, we provide numeric morphology-based evidence that the dark-colored Poneracoarctatavar.lucida Emery, 1898, formerly considered a synonym of *P.coarctata* (Latreille, 1802), is conspecific with the lighter-colored *Poneratestacea* Emery, 1895. Species hypotheses are developed via NC-PART clustering, combined with Partitioning Algorithm based on Recursive Thresholding (PART), and via PCA combined with gap statistics. We obtained our results from an extensive dataset from the 10 continuous morphometric traits measured on 165 workers belonging to 73 nest samples. Linear discriminant analysis (LDA) confirmed the grouping of hypotheses generated by exploratory analyses with 100% classification success when all ten morphometric traits were involved. The Anatolian Turkish black and the predominantly European yellow samples, did not separate based on their morphometric characteristics. These two color variations broadly overlap in their geographic range in Anatolian Turkey. The investigated type series of Poneracoarctatavar.lucida Emery, 1898 (collected from Kazakhstan) fell within the *P.testacea* cluster instead of *P.coarctata* and is also classified with high certainty as *P.testacea* by confirmatory LDA. Therefore, we propose the synonymy of Poneracoarctatavar.lucida Emery, 1898 with *Poneratestacea* Emery, 1895. As no other morphological differences than color patterns were detected between the “black” and “pale” *P.testacea* samples, we hold that these populations constitute geographically occurring color variations of the same species. Finally, our quantitative morphology-based results show that relying on color patterns is not a robust approach in identifying European *Ponera* samples, particularly in the east, but using multivariate analyses of morphometric traits is advised instead.

## ﻿Introduction

The taxonomy of the European representatives of the tiny hypogeic genus *Ponera* has for several decades been apparently unambiguous. The two European species belonging to this genus, *P.coarctata* (Latreille, 1802) and *P.testacea* Emery, 1895 constitute one of the continent’s most easily distinguishable species pairs (Csősz and Seifert 2003; Scupola 2006; Czechowski and Radchenko 2010; Attewell et al. 2011). Conspicuous size and color features help to tell these species apart. *Poneracoarctata* is larger, generally black, and has a higher petiole, while its typically lighter yellow congener, *P.testacea*, is significantly smaller, having a low and thick petiolar node (Csősz and Seifert 2003). In addition, these species differ in habitat preferences; *P.testacea* prefers more xerothermous biotopes than its sister species, *P.coarctata*. Based on several environmental variables measured in 25 study plots in Central Europe (Seifert 2017), *P.testacea* sites can be characterized as having higher maximum calibrated soil temperatures and lower soil moisture values. These previously considered European species extend their distribution to Turkey (Kiran and Karaman 2021), and *P.coarctata* has been known to occur north of the Black Sea coastline and reach the Caucasus range (Kiran and Karaman 2020). However, the most recent investigations revealed that a third morph, with a mixed combination of traits, also appears to occur in this region. This form is similar to *P.coarctata* in its shiny black or dark brown color. At the same time, the lower petiole node, dense pilosity, and multivariate analyses on their morphometric data place representatives of this particular group in *P.testacea*. Therefore, the normal morphological approaches that help to separate these *Ponera* species in the rest of the European part of the Western Palaearctic region seemed to fail in accurately identifying them in Turkey.

This problem prompted us to examine the possibility of whether a third *Ponera* species appeared in this region and, if so, to describe it appropriately. Ant taxonomists have often considered color traits unreliable as species-level traits due to the high intraspecific color polymorphism (Seifert 2003a,b, 2019; Seifert et al. 2017) that may reportedly lead to taxonomic errors. Therefore, we analyzed an extensive set of continuous morphometric data via robust multivariate statistic procedures, NC-clustering and principal component analysis (PCA) to infer species boundaries. Both these approaches were used in combination with Gap statistic algorithms that estimate the number of clusters in the data and assign observations (i.e. specimens or samples) into partitions. We tested the validity of the recognized pattern via confirmatory linear discriminant analysis (LDA).

We compared the morphologically recognized clusters with the color patterns of the samples. As a result, the third *Ponera* morph found in Turkey broadly overlaps with the cluster of European *P.testacea* via the complex morphometric protocol. The Anatolian Turkish black and the predominantly European yellow samples did not separate on the basis of their morphometric characteristics, and these two color variations broadly overlap in their geographic range in Anatolian Turkey. The investigated type series of Poneracoarctatavar.lucida Emery, 1898 (collected from Azerbaijan) fell within the *P.testacea* cluster instead of *P.coarctata* and is also classified with high certainty as *P.testacea* by confirmatory LDA. Therefore, we propose the synonymy of *Poneralucida* with *Poneratestacea*. As no other morphological differences than color patterns were detected between the “black” and “pale” *P.testacea* samples, we hold that these populations constitute geographically occurring color variations of the same species. Thus, making *P.lucida* a subspecies of *P.testacea* is proven unnecessary.

## ﻿Material and methods

In this research 10 continuous morphometric traits were measured on 165 workers belonging to 73 nest samples. The material is deposited in the following institutions: Entomological Museum of Trakya University (EMTU), Hungarian Natural History Museum, Budapest, Hungary (HNHM), Muséum d’histoire naturelle, Genève (MHNG), Natural History Museum, Genoa (MSNG), private collection of Sándor Csősz (PCSC). The full list of material investigated is listed below:

### ﻿Material examined

#### Type material

**Poneracoarctatavar.testacea Emery, 1895**

**Lectotype**: “Bonifacio, leg. REVEL 1872” (1 w, MCSN); paralectotypes (3 workers) Rapallo / Liguria / Mai 1891” (1 w, MHNG); and two other specimens (on one pin) labelled by Emery “Gallia merid.” and with a blue label “Cotypus” (2 ww, MHNG)

**Poneracoarctatavar.lucida Emery, 1898**

**Syntypes**: “Lenkoran / (next line) Korb / next label Syntypus *Poneracoarctata* var. lucida Emery, 1898” (3 ww), MCSN) [1^st^ and 3^rd^ measured, 2^nd^ not measurable due to glue obstructing the view]

#### Other material

***Poneracoarctata***

Austria: **AUT:Felsolovo**: Felsőlövő, 1911.04.03, N47.35, E16.20, (3, HNHM); Croatia: **CRO:Buccari**: Buccari, 1927.04.21, N45.30, E14.53, (3, HNHM); **CRO:Orehovica**: Orehovica, 1885.06.04, N46.33, E16.50, (5, HNHM); Hungary: **HUN:Badacsony**: Badacsony, 1929.08.24, N46.79, E17.50, (9, HNHM); **HUN:Batorliget**: Bátorliget, 1948.06.17, N47.76, E22.27, (6, HNHM); **HUN:Kolked**: Kölked, 1924.05.23, N45.95, E18.70, (3, HNHM); **HUN:Mezokovacshaza**: Mezőkovácsháza, 1886.07.15, N46.40, E20.90, (2, HNHM); **HUN:Nagyvazsony**: Nagyvázsony, Kab-hegy, 1924.05.06, N47.046, E17.65, (5, HNHM); **HUN:Simontornya**: Simontornya, 1913.06.18, N46.75, E18.55, (6, HNHM); Montenegro: **MNE:Zlatitca**: Zlatitca, 1886.06.07, N 42.46, E 19.29, (5, HNHM); Romania: **ROM:O.Sebeshely**: O.Sebeshely, 1913.07.03, N45.75, E23.25, (1, HNHM); **ROM:Tasnad-Szarvad**: Tasnád Szarvad, 1882.12.12, N47.47, E22.58, (3, HNHM); Slovakia: **SLO:Baan**: Baán [Bánovce], 1881.08.22, N48.72, E18.26, (3, HNHM); Switzerland: **SWI:Enge**: Enge, 1880.04.12, N47.36, E8.53, (1, MHNG); **SWI:Neuveville**: Neuveville, N47.063, E7.091, (7, MHNG); Turkey: **TUR:10/1520**:**Çorum**: Uğurludağ, 2010.06.15, N40.3864, E35.4650, (2, EMTU); **TUR:06/035**: **Edirne**: 2006.09.13, N41.67, E26.55, (2, EMTU); **TUR:12/719a**:**Giresun**: Bulancak-Tekmezar Vill., 2012.06.12, N40.8556, E38.2403, (1, EMTU); **TUR:12/721**: Bulancak-Tekmezar Vill., 2012.06.12, N40.8556, E38.2403, (1, EMTU); **TUR:12/931a**: Espiye-Çepni Vill., 2012.06.15, N40.8575, E38.7314, (2, EMTU); **TUR:12/615b**: Piraziz-Armutçukuru Vill., 2012.06.11, N40.8367, E38.08001, (2, EMTU); **TUR:12/626**: Piraziz-Armutçukuru Vill., 2012.06.11, N40.8367, E38.0800, (2, EMTU); **TUR:11/197b**:**Kırklareli**: Demirköy-Sivriler Vill., 2011.05.24, N41.78, E27.86, (2, EMTU); **TUR:11/199a**: Demirköy-Sivriler Vill., 2011.05.24, N41.7839, E27.8651, (2, EMTU); **TUR:11/0203**: Demirköy-Sivriler Vill., 2011.05.24, N41.78, E27.86, (2, EMTU); **TUR:11/0253**: Pınarhisar-Yenice Vill., 2011.05.25, N41.7408, E27.7114, (2, EMTU); **TUR:11/157a**: Vize-Kıyıköy, 2011.05.22, N41.6345, E28.0697, (2, EMTU); **TUR:12/0539a**:**Ordu**: Şenocak Vill., 2012.06.10, N40.8867, E37.9567, (2, EMTU); **TUR:12/542b**: Şenocak Vill., 2012.06.10, N40.8867, E37.9567, (2, EMTU); **TUR:12/553a**: Kabadüz-Harami Vill., 2012.06.10, N40.8242, E37.9258, (2, EMTU); Ukraine: **UKR:Beregszasz**: Beregszász, 1883.10.15, N48.20, E22.63, (1, HNHM);

***Poneratestacea***

“BLACK MORPH”

**AZE:Lenkaran-lucida-ST**: Lenkoran [Lankaran, syntype series], Korb, N38.75, E48.85, (2, MSNG); **TUR:07/2463**:**Balıkesir**: Bayramiç-Adalı Vill., 2007.09.07, N39.3697, E28.2811, (1, EMTU); **TUR:10/726:Çankırı**: Yapraklı-Ayseki Vill., 2010.06.01, N40.7925, E33.9014, (2, EMTU); **TUR:12/1109b**:**Gümüşhane**: Kürtün-Taşlıca Vill., 2012.06.17, N40.7187, E39.0344, (2, EMTU); **TUR:K98/483a**:**İzmit**: Karamürsel-Tahtalı Vill., 1998.08.05, N40.5775, E29.6441, (1, EMTU); **TUR:04/914a**: Derbent-Sultaniye Vill., 2004.08.29, N40.6106, E30.0867, (2, EMTU); **TUR:04/915b**: Derbent-Sultaniye Vill., 2004.08.29, N40.6106, E30.0867, (2, EMTU); **TUR:04/796**:**Konya**: Altınopa Dam Lake, 2006.08.27, N37.88, E32.29, (1, EMTU); **TUR:11/680**: Akşehir-Engili Vill., 2011.06.27, N38.3031, E31.4467, (2, EMTU); **TUR:12/553b**:**Ordu**: Kabadüz-Harami Vill., 2012.06.10, N40.8242, E37.9258, (2, EMTU); **TUR:12/1941a**:**Rize**: Çamlıhemşin-Topluca Vill., 2012.08.05, N41.0603, E41.0158, (1, EMTU); **TUR:12/2352**: Ardeşen-Sinan Vill., 2012.08.07, N41.0930, E41.0895, (2, EMTU); **TUR:10/1754a**:**Sivas**: Gürün-Reşadiye Vill., 2010.08.14, N38.8214, E37.1892, (2, EMTU); **TUR:12/1326**:**Trabzon**: Düzköy-Aykut, 2012.06.20, N40.9122, E39.4581, (2, EMTU); **TUR:12/1415b**: Maçka-Acısu Vill., 2012.06.21, N40.7125, E39.5920, (2, EMTU); **TUR:12/1416**: Maçka-Acısu Vill., 2012.06.21, N40.7125, E39.5919, (2, EMTU); **TUR: 12/1421b**: Maçka-Akmescit Vill., 2012.06.21, N40.8405, E39.6547, (2, EMTU); **TUR:K98/656**:**Yalova**: Armutlu-Hayriye Vill., 1998.08.14, N40.5008, E28.9664, (2, EMTU).

“PALE MORPH”

**CRO:Senj**: Zengg [Senj], N44.9893, E14.9037, (1, HNHM); **FRA:Corse-Bonifacio(LT)**: Bonifacio [lectotype], 1972, N41.39, E9.159, (1, MSNG); **FRA:Gallia meridionale (PLT)**: Gallia Meridionale [paralectotype], (2, MSNG); **HUN:Pusztapoo**: Pusztapoó, 1929.02.11, N47.093, E20.4556, (6, HNHM); **HUN:Rimaszombat**: Rimaszombat, 1909.07.10, N48.382, E20.021, (2, HNHM); **HUN:Szigetszentmiklos**: Szigetszentmiklós, 1912.10.16, N47.34, E19.035, (2, HNHM); **ITA:Rapallo (PLT)**: Rapallo [paralectotype], 1891.05, N4435, E9.23, (1, MHNG); **TUR:12/2674**:**Artvin**: Şavşat-Maden Vill., 2012.08.11, N41.3781, E42.1333, (2, EMTU); **TUR:K98/0689a**:**Bilecik**: Osmaneli-Yeşilçimen Vill., 1998.08.17, N40.4475, E29.8881, (2, EMTU); **TUR:10/1515b**:**Çorum**: Uğurludağ, 2010.06.15, N40.3863, E35.465, (2, EMTU); **TUR:10/1520b**: Uğurludağ, 2010.06.15, N40.3863, E35.4650, (2, EMTU); **TUR:12/929**:**Giresun**: Espiye-Çepni Vill., 2012.06.15, N40.8575, E38.7314, (1, EMTU); **TUR:12/1071**: Tirebolu, 2012.06.16, N41.0217, E38.8942, (1, EMTU); **TUR:10/1269b**:**Kayseri**: Pınarbaşı-Cinliyurt Vill., 2010.06.09, N38.5019, E36.1909, (2, EMTU); **TUR:10/1278b**: Pınarbaşı-Cinliyurt Vill., 2010.06.09, N38.5019, E36.1908, (2, EMTU); **TUR:10/1285**: Melikgazi, 2010.06.09, N38.7195, E36.2142, (1, EMTU); **TUR:10/1286**: Melikgazi, 2010.06.09, N38.7195, E36.2141, (1, EMTU); **TUR:11/231a**:**Kırklareli**: Vize, 2011.05.25, N41.5837, E27.2703, (1, EMTU); **TUR:11/232a**: Vize, 2011.05.25, N41.5836, E27.7102, (2, EMTU); **TUR:12/1831a**:**Rize**: Pazar-Örnek Vill., 2012.08.03, N41.1464, E40.7978, (2, EMTU); **TUR:10/766**:**Yozgat**: Sungurlu-Çingirler Vill., 2010.06.02, N40.2814, E34.37, (2, EMTU).

Distribution map for all species discussed in this revisionary work is generated via SimpleMappr (Shorthouse 2010).

### ﻿Protocol for color-coding

**Figure 1.**
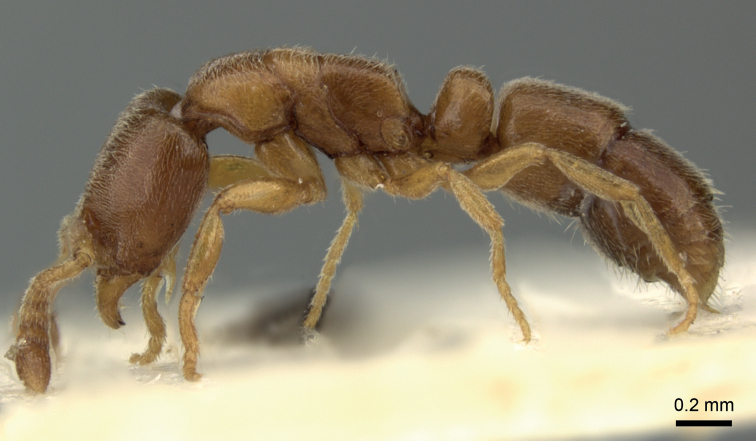
Light-colored (score 2) *Poneratestacea* worker from Hungary. Specimen: CASENT0906719, from www.antweb.org.

**Figure 2.**
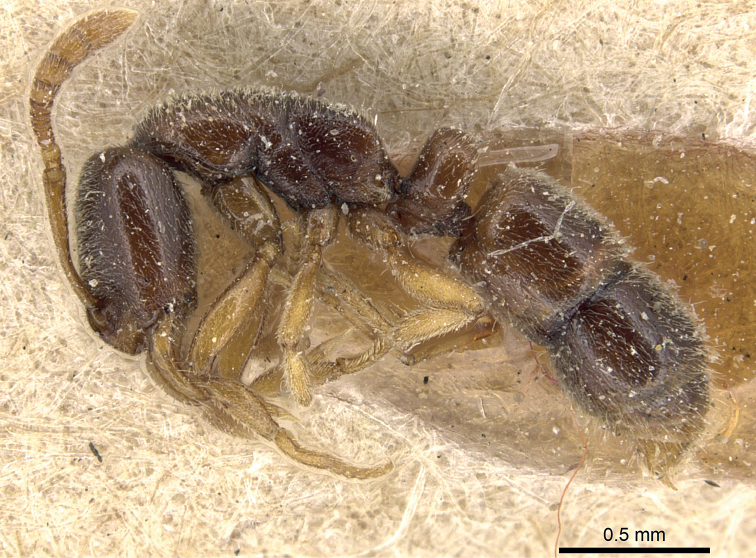
A syntype worker of Poneracoarctatavar.lucida representing a dark-colored (score 4) *P.testacea* worker from Azerbaijan. Specimen: CASENT0903905, from www.antweb.org.

Pigmentation scaling was performed via a subjective evaluation of body coloration ranging from whitish yellow (score 1) to black (score 5). The specimens were illuminated via Photonic Optics 2-arms Illuminator with neutral white color temperature, 5900 K (equivalent to halogen, 4000 K). Specimens with light pigmentation (score 2 and score 3) were classified as “pale morph” (Fig. 1.), darker (score 4 and score 5) specimens were considered “black morph” (Fig. 2). Very light, whitish-yellow (score 1) specimens have not been found.

### ﻿Protocol for morphometric character recording

Morphometric characters are defined as in Seifert (2018). All measurements were made in an Olympus SZX9 stereomicroscope at a magnification of 150× for each character. Morphometric data are given in µm throughout the paper. All worker individuals were measured by SC. Definitions of morphometric characters are listed below (for details see Csősz and Seifert 2003: 202–204):

CL Cephalic length;

CW Cephalic width;

FL Maximum width of frontal lobes;

FR Minimum distance between frontal carinae;

ML Mesosoma length;

PEH The maximum height of petiole;

PEL Petiole length;

PH Height of petiole node;

PW Petiole width;

SL Scape length.

### ﻿Statistical framework on morphometric data–hypothesis formation and testing

#### Exploratory analyses through NC-PART clustering

The statistical procedure has been done on worker caste only. The prior species hypothesis was generated based on workers through the combined application of NC clustering (Seifert et al. 2014) and Partitioning Algorithm based on Recursive Thresholding (PART) (Nilsen et al. 2013; see also Csősz and Fisher 2016). The optimal number of clusters and the partitioning of samples are accepted as the preliminary species hypothesis in every case in which the two clustering methods, ‘hclust’ and ‘kmeans’ through PART, have yielded the same conclusion.

#### Exploratory analyses through sPCA in combination with Gap statistics

Structure in morphometric data was also displayed in a scatterplot via a principal component analysis (sPCA; Baur and Leuenberger 2011). The sPCA does not return an estimate on the number of clusters, hence an iterative gap statistic algorithm (function ‘gap’) implemented in package clusterGenomics (Tibshirani et al. 2001; Nilsen et al. 2013), was employed to determine the optimal number of clusters within data and also assigned observations (i.e. specimens, or samples) into partitions.

#### Hypothesis testing by confirmatory analyses

The validity of the prior species hypothesis imposed by the two exploratory processes was tested via a cross-validated linear discriminant analysis (CV-LDA), and the best fitting simple ratio is found via multivariate ratio analysis (MRA). Statistical analyses have been done in R (R Core Team 2019).

## ﻿Results

### ﻿Finding biodiversity patterns through numeric morphology

**Figure 3.**
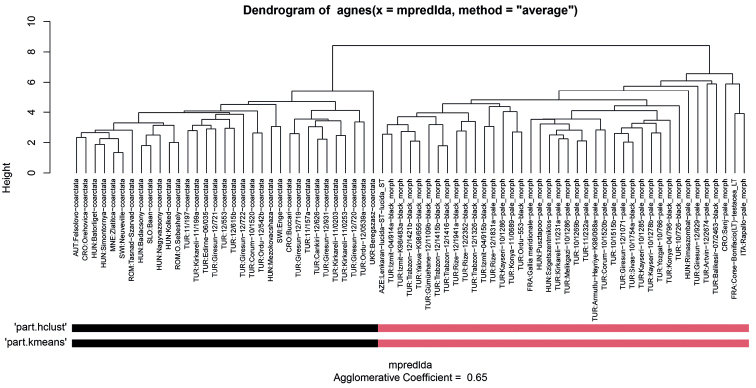
Dendrogram solution for the Western Palaearctic representatives of *Ponera*. Sample information in the dendrogram follows this format: abbreviated country code, locality name, and/or a special collection code followed by final species hypothesis separated by underscore. Two columns of rectangles represent results of partitioning resulted by method PART using two cluster methods ‘hclust’ and ‘kmeans’.

**Figure 4.**
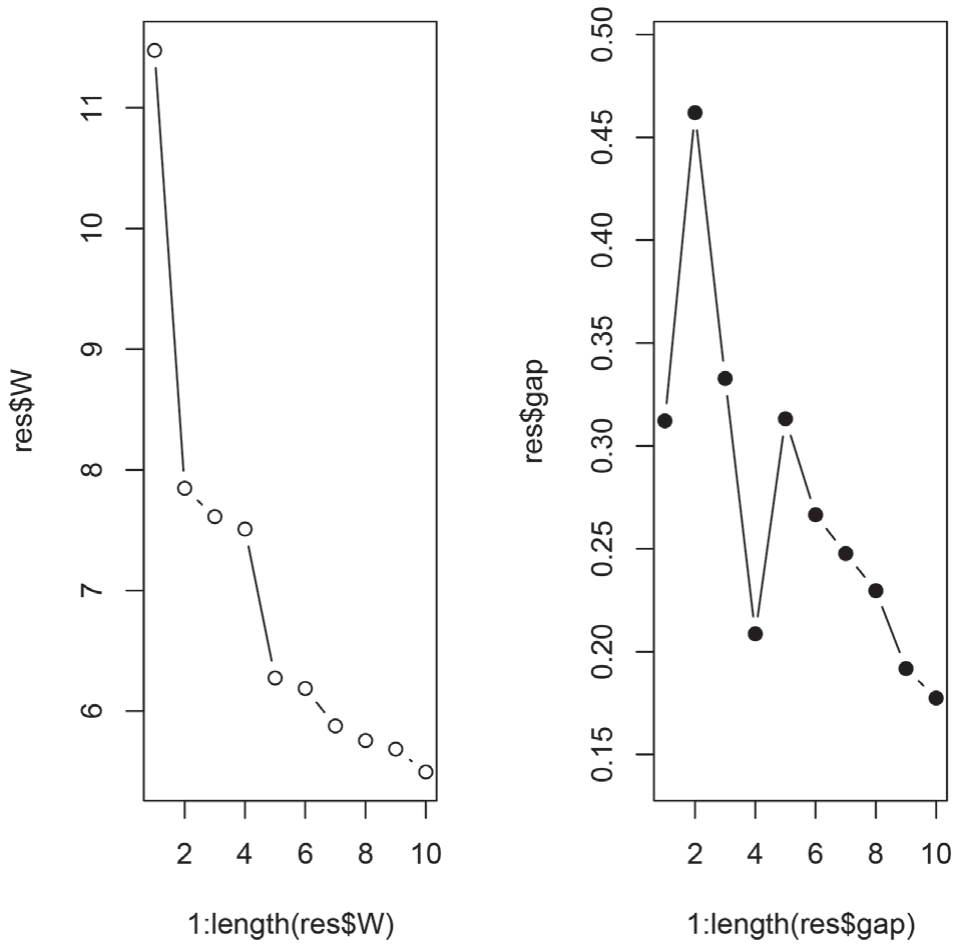
Gap statistic for dataset of Western Palaearctic *Ponera* samples. Two-cluster solution is highly supported by the elbow at 2 components by the dispersion curve (left) and by the peak at cluster number four by the gap curve (right). Number of clusters in the data (X axis), the total within-cluster dispersion for each evaluated partition (Y axis for the left plot) and the vector of length Kmax giving the Gap statistic for each evaluated partition (Y axis for the right plot) is illustrated.

**Figure 5.**
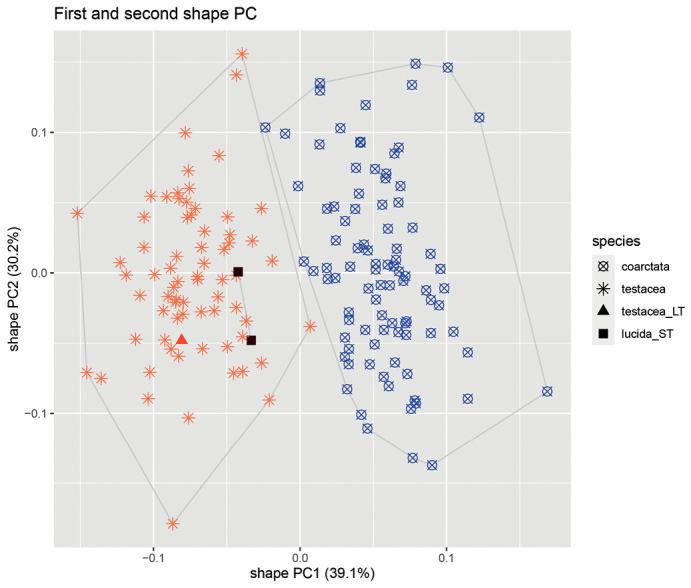
Ordination biplot for shape principal component analysis (sPCA) based on species identity. Color codes represent: *Poneracoarctata*: blue circles, *P.testacea*: orange asterisks, *P.testacea* lectotype: orange triangle, *P.lucida* syntypes: dark brown rectangles.

Altogether two clusters were revealed to be the most parsimonious solution by both NC-PART clustering (Fig. 3.) and by the gap statistic based on PC scores (Figs 4, 5.). The grouping hypotheses generated by hypothesis-free exploratory analyses is confirmed by LDA with 100% classification success when all 10 morphometric traits were involved. This pattern is also supported by the examination of external morphological traits (e.g. shape of petiolar node, and density of pilosity on the first gaster tegite). *Poneracoarctata* appears to exhibit uniform color patterns throughout the whole distributional area, but *P.testacea* has a rather bimodal coloration; the western (European) population has a pale brown, or yellow appearance, whilst the eastern, Anatolian samples are black. This geography driven shift does not appear in other morphological features and the multivariate analyses of continuous traits do not support separateness.

### ﻿Taxonomic acts

We hold *P.testacea* does not split into separate subspecies and the “pale morph” and the “black morph” of *P.testacea* belong to the same species. Therefore, we synonymize Poneracoarctatavar.lucida Emery, 1898, which was formerly considered a junior synonym of *P.coarctata* (see Taylor 1967), with *P.testacea* Emery, 1895.

### ﻿The spatial distribution pattern

Geographic distribution of *Poneracoarctata* and *P.testacea* (including the “pale morph” and the “black morph”) broadly overlap in Europe and in Anatolian Turkey, where *P.coarctata* occupies significantly (*p* = 0.046) lower altitudes (519 m [5 m, 743 m]) than *P.testacea* (900 m [0 m, 1900 m]). The two color variations of *P.testacea* does not show significant differences (*p* = 0.92) in their vertical distribution (“black morph” (*n* = 14) 896 m [442 m, 1791 m], “pale morph” (*n* = 10) 919 m [0 m, 1900 m]).

### ﻿Species delimitation

**Figure 6.**
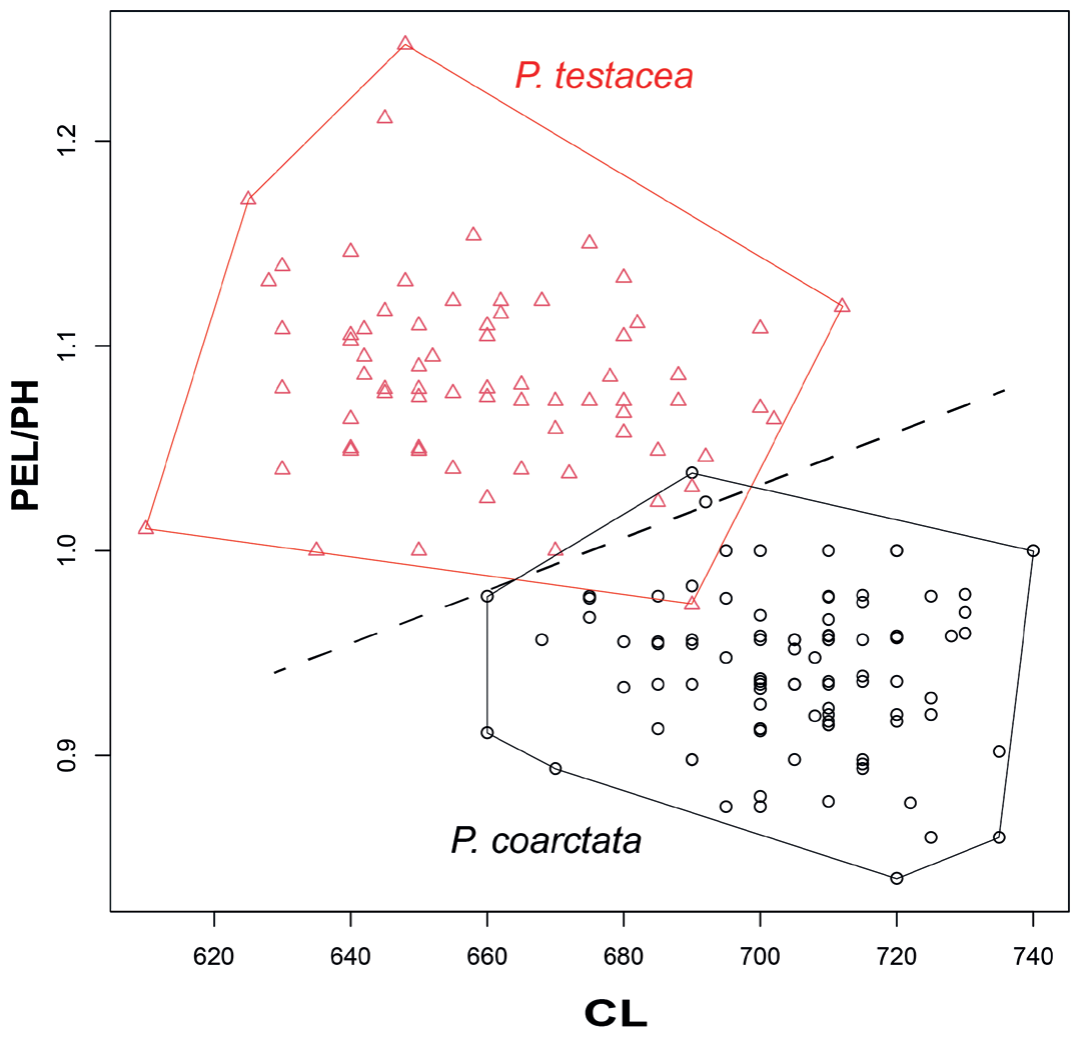
The best morphometric ratio (petiole length / petiole height; PEL/PH) is illustrated on the head length (CL). Scatterplots of the most discriminating ratio on the head length between workers of Western Palaearctic representatives of *Ponera*; *P.coarctata*: black circles, *P.testacea*: red triangles. The thin dashed line illustrates best separation.

The distinctive morphology of these species allows for considerable reduction of morphological characters, so that workers of the two taxa *Poneracoarctata* and *P.testacea* can be separated based on the combination of three continuous morphometric traits (CW, PEL, and PH; Fig. 6) with 99.4% (a single misclassified case out of the total 165 individuals).

Coefficients of linear discriminants help to place and identify samples via placing workers in the discriminant space using the linear discriminant function (LD) as follows (morphometric traits are in micrometers):

LD = 0.0449*CW − 0.0893 * PEL + 0.0750 * PH − 21.4786

*Poneracoarctata* (*n* = 93) = +1.99 (+0.22, +5.02)

*Poneratestacea* (*n* = 72) = −2.57 (−5.05, +0.27) (including both “pale morph” and “black morph”)

A simple morphometric ratio of two petiole characters (petiole length / height of petiolar node, PEL/PH) appears an excellent numeric key to tell these species apart but is slightly affected by allometry. Therefore, a graphical display of this ratio on the head length (CL) is also provided as an easy-to-use asset aiding routine determinations (Fig. 6). This key yields a 98.18% classification success (*n* = 165).

## ﻿Discussion

The yellow and black *Poneratestacea* morphs do not differ via multivariate analyses of continuous morphometric traits based on an extensive material collected in a wide geographic range. Furthermore, no other shape characteristics support their separation. The investigated type series of Poneracoarctatavar.lucida (the “black” morph) fell within the *P.testacea* cluster instead of *P.coarctata* in both exploratory analyses (NC-clustering and PCA) and is also classified with high certainty as *P.testacea* by confirmatory LDA. Therefore, we propose synonymy of *Poneralucida* with *Poneratestacea* instead of *P.coarctata*.

**Figure 7.**
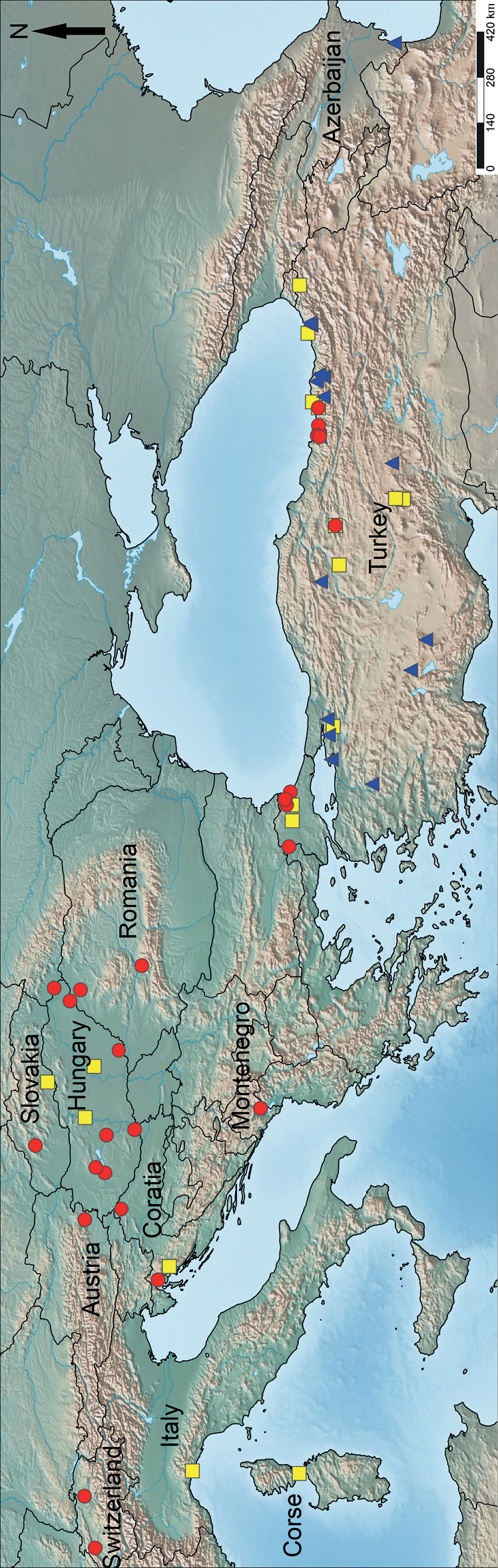
Geographic map of *Ponera* species in Europe and Turkey. Color codes for species are as follows: *Poneracoarctata*: red circles, *P.testacea* “black morphs”: blue triangles, *P.testacea* “yellow morphs”: yellow rectangles.

Although the yellowish phenotype of *Poneratestacea* is dominant in the Western Palaearctic (western Turkey and Europe), the black *P.testacea* morph is prevalent in Turkey; distribution of these color variations broadly overlaps in Anatolia (Fig. 7). The sympatric pattern might indicate a subspecific rank. However, due to the lack of morphological differences other than the color of *P.testacea* morphs, we hold that these populations constitute geographically occurring color variations of the same species, making *P.lucida* a subspecies of *P.testacea* unnecessary.

In conclusion, relying solely on color patterns is not a robust approach in identifying European *Ponera* samples, but using multivariate analyses of morphometric traits or the presented numeric key is advised to distinguish these species. Straightforward pigmentation patterns, such as variation of light versus dark, red versus brown, or red versus black on the whole body or specific body parts, are frequently unreliable taxonomic characters in insects and other animals. A minor point mutation may change pigmentation profoundly, whereas complex morphological structures are less easily changed. Many textbook examples demonstrate relatively simple pigmentation genetics (e.g. Lus 1932; Barrion and Saxena 1987; Majerus 1998; Andrés and Cordero 1999; Majumdar et al. 2008). Taxonomic failures or pitfalls resulting from pigmentation in ants have been repeatedly reported. Seifert (1997, 2018, 2020) described color dimorphism as superimposed by an allometric change in the *Formicarufibarbis* group and *Lasius* species. Intraspecific color polymorphism superimposed by geographic clines has also been shown for species of the *Formicacinerea* group (Seifert 2003a). Furthermore, extreme intraspecific color polymorphism is reported in several species of *Cardiocondyla* (Seifert 2003b; Seifert et al. 2017) and in *Camponotus* (Seifert 2019) that reportedly leads to taxonomic errors.

## References

[B1] Andrés JA Cordero A (1999) The inheritance of female color morphs in the damselfly *Ceriagriontenellum* (Odonata, Coenagrionidae). Heredity 82: 328–335. 10.1038/sj.hdy.6884930 10336708

[B2] AntWeb (2020) AntWeb. Version 8.68.7. California Academy of Science. Accessed on: 2022-1-5. https://www.antweb.org

[B3] Attewell PJ Collingwood CA Godfrey A (2010) *Poneratestacea* (Emery, 1895) (Hym.: Formicidae) new to Britain from Dungeness, East Kent. Entomologist’s Record and Journal of Variation 122: 113–119.

[B4] Barrion AA Saxena RC (1987) Inheritance of body color in the brown planthopper, *Nilaparvatalugens*. Entomologia Experimentalis et Applicata 43: 267–270. 10.1111/j.1570-7458.1987.tb02220.x

[B5] Baur H Leuenberger C (2011) Analysis of ratios in multivariate morphometry. Systematic Biology 60(6): 813–825. 10.1093/sysbio/syr061 21828084 PMC3193766

[B6] Csősz S Fisher BL (2016) Taxonomic revision of the Malagasy members of the *Nesomyrmexangulatus* species group using the automated morphological species delineation protocol NC-PART-clustering. PeerJ 4: e1796. 10.7717/peerj.1796 PMC4793320 26989630

[B7] Csősz S Seifert B (2003) *Poneratestacea* Emery, 1895 stat. n.—a sister species of *P.coarctata* (Latreille, 1802) (Hymenoptera: Formicidae). Acta Zoologica Academiae Scientiarum Hungaricae 49(3): 211–223.

[B8] Czechowski W Radchenko A (2010) *Poneratestacea* Emery, 1895 (Hymenoptera: Formicidae) in Poland. Polish Journal of Entomology 79: 327–337.

[B9] Kiran K Karaman C (2020) Additions to the ant fauna of Turkey (Hymenoptera, Formicidae). Zoosystema 42(18): 285–329. 10.5252/zoosystema2020v42a18

[B10] Kiran K Karaman C (2021) Ant fauna (Hymenoptera: Formicidae) of Central Anatolian Region of Turkey. Turkish Journal of Zoology 45(3): 161–196. 10.3906/zoo-2008-6

[B11] Lus JJ (1932) An analysis of the dominance phenomenon in the inheritance of the elytra and pronotum color in *Adaliabipunctata*. Trudy Labroratorii Genetiki, Leningrad 9: 135–162.

[B12] Majerus M (1998) Melanism: Evolution in Action. Blackwell, Oxford, 338 pp.

[B13] Majumdar KC Nasuruddin K Ravinder K (2008) Pink body color in *Tilapia* shows single gene inheritance. Aquaculture Research 28(8): 581–589. 10.1046/j.1365-2109.1997.00898.x

[B14] Nilsen G Borgan Ø LiestØl K Lingjaerde OC (2013) Identifying clusters in genomics data by recursive partitioning. R package version 1.0. 10.1515/sagmb-2013-0016 23942354

[B15] R Core Team (2019) R: A language and environment for statistical computing. R Foundation for Statistical Computing, Vienna, Austria. https://www.R-project.org/

[B16] Scupola A (2006) Poneracoarctatavar.crassisquama Emery, 1916 a new synonym of *P.testacea* Emery, 1895 (Hymenoptera, Formicidae). Bollettino del Museo Civico di Storia Naturale di Verona Botanica Zoologia 30: 161–164.

[B17] Seifert B (1997) *Formicalusatica* n. sp.—a sympatric sibling species of *Formicacunicularia* and *Formicarufibarbis* (HymenopteraFormicidae). Abhandlungen und Berichte des Naturkundemuseums Görlitz 69(5): 3–16.

[B18] Seifert B (2003a) A taxonomic revision of the *Formicacinerea* group (Hymenoptera: Formicidae). Abhandlungen und Berichte des Naturkundemuseums Görlitz 74(2): 245–272.

[B19] Seifert B (2003b) The ant genus *Cardiocondyla* (Insecta: Hymenoptera: Formicidae)—a taxonomic revision of the *C.elegans*, *C.bulgarica*, *C.batesii*, *C.nuda*, *C.shuckardi*, *C.stambuloffii*, *C.wroughtonii*, *C.emeryi*, and *C.minutior* species groups. Annalen des Naturhistorischen Museums Wien, Serie B 104: 203–338.

[B20] Seifert B (2017) The ecology of Central European non-arboreal ants—37 years of a broad-spectrum analysis under permanent taxonomic control. Soil Organisms 89(1): 1–69.

[B21] Seifert B (2018) The Ants of Central and North Europe. Lutra Verlags- und Vertriebsgesellschaft, Tauer, 408 pp.

[B22] Seifert B (2019) A taxonomic revision of the members of the *Camponotuslateralis* species group (Hymenoptera: Formicidae) from Europe, Asia Minor and Caucasia. Soil Organisms 91(1): 7–32. 10.25674/so-91-1-02

[B23] Seifert B (2020) A taxonomic revision of the Palaearctic members of the subgenus Lasius s.str. (Hymenoptera, Formicidae). Soil Organisms 92(1): 15–86. 10.25674/so92iss1pp15

[B24] Seifert B Okita I Heinze J (2017) A taxonomic revision of the *Cardiocondylanuda* group (Hymenoptera: Formicidae). Zootaxa 4290(2): 324–356. 10.11646/zootaxa.4290.2.4

[B25] Seifert B Ritz M Csősz S (2014) Application of exploratory data analyses opens a new perspective in morphology-based alpha-taxonomy of eusocial organisms. Myrmecological News 19: 1–15.

[B26] Shorthouse DP (2010) SimpleMappr, an online tool to produce publication-quality point maps. Accessed on: 2021-11-18. https://www.simplemappr.net

[B27] Taylor RW (1967) A monographic revision of the ant genus *Ponera* Latreille (Hymenoptera: Formicidae). Pacific Insects Monograph 13: 1–112.

[B28] Tibshirani R Walther G Hastie T (2001) Estimating the number of clusters in a data set via the gap statistic. Journal of the Royal Statistical Society: Series B (Statistical Methodology) 63(2): 411–423. 10.1111/1467-9868.00293

